# The Effect of Curative Treatment on Hyperglycemia in Patients With Cushing Syndrome

**DOI:** 10.1210/jendso/bvab169

**Published:** 2021-12-02

**Authors:** Justine Herndon, Ravinder Jeet Kaur, Mark Romportl, Emily Smith, Amy Koenigs, Brenda Partlow, Leonardo Arteaga, Irina Bancos

**Affiliations:** 1 Division of Endocrinology, Diabetes and Nutrition, Mayo Clinic, Rochester, Minnesota 55905, USA; 2 Division of Endocrine Research, Mayo Clinic, Rochester, Minnesota 55905, USA; 3 Department of Laboratory Medicine and Pathology, Mayo Clinic, Rochester, Minnesota 55905, USA

**Keywords:** HbA_1c_, diabetes mellitus, hypercortisolism, improvement, outcome, surgery

## Abstract

**Context:**

Hyperglycemia is a common complication of Cushing syndrome (CS).

**Objective:**

We aimed to determine the impact of curative procedure on hyperglycemia and its management in patients with CS.

**Methods:**

This retrospective longitudinal cohort study took place 2000 to 2019 in a referral center among adults with endogenous CS and hyperglycemia. Main outcome measures included glycated hemoglobin A_1c_ (HbA1c), intensity of hyperglycemia therapy, and improvement of hyperglycemia.

**Results:**

In 174 patients with CS (pituitary in 106, ectopic in 25, adrenal in 43), baseline median HbA_1c_ was 6.9% (range, 4.9-13.1), with 41 (24%) patients not on any therapy for hyperglycemia, 93 (52%) on oral medications, and 64 (37%) on insulin (median daily units of 58; range, 10-360). Following CS remission, at the end of follow-up (median 10.5 months), 37 (21%) patients demonstrated resolution of hyperglycemia, 82 (47%) demonstrated improvement, and 55 (32%) had no change or worsening in hyperglycemia. At the end of follow-up, HbA_1c_ decreased by 0.84% (*P* < .001) and daily insulin dose decreased by a mean of 30 units (*P* < .001). Biochemical hypercortisolism severity score (severe vs moderate/mild: odds ratio [OR] of 2.4 [95% CI, 1.1-4.9]), and CS subtype (nonadrenal vs adrenal: OR of 2.9 [95% CI, 1.3-6.4]), but not type of hyperglycemia (diabetes vs prediabetes: OR of 2.1 [0.9-4.9]) were associated with hyperglycemia improvement at the end of follow-up.

**Conclusion:**

Two-thirds of patients with CS and hyperglycemia demonstrate resolution or improvement of hyperglycemia after a curative procedure. Close monitoring during CS recovery is needed to ensure appropriate therapy modification.

Cushing syndrome (CS) develops in patients with chronic exposure to endogenous or exogenous cortisol excess [1]. Endogenous CS can be divided into adrenocorticotropin (ACTH)-dependent (pituitary and ectopic) or ACTH-independent (adrenal) subtypes. CS is associated with a higher risk of cardiometabolic conditions, bone disease, coagulopathy, and infections [2-4]. Hyperglycemia is frequently associated with CS [5-7]. Chronic hypercortisolism leads to hyperglycemia through several mechanisms that include exacerbation of the liver gluconeogenesis, reduction of glycogen synthesis, decrease in insulin sensitivity and β-cell function in the pancreas, and impairment of the glucose uptake to the muscle [5, 8-13].

Diabetes mellitus (DM) has been reported in up to 20% to 48% of patients with CS, and hyperglycemia (DM and prediabetes) in up to 38% to 84% of patients with CS [2, 5, 6]. In patients with mild cortisol excess, such as those with adrenal adenomas and abnormal dexamethasone suppression test (mild autonomous cortisol secretion), prevalence of DM has been reported to be 28% [7]. Notably, studies have varied in how hyperglycemia was assessed and whether the assessment was uniform among patients. In addition, patient populations varied regarding the CS subtype, duration of CS, and severity of hypercortisolism, further contributing to the wide spectrum of estimates. Several studies reported on the effect of a curative surgery for CS on hyperglycemia. Studies mainly addressed hyperglycemia improvement in patients with pituitary CS [14-21], with only a few small studies including ectopic [15, 16, 22] and adrenal CS [14, 16, 22]. Most studies reported on the prevalence of hyperglycemia at baseline and follow-up, but did not distinguish between resolution or improvement in hyperglycemia and whether the outcomes differed based on the severity and subtype of CS. In addition, no studies reported on the degree of hyperglycemia improvement or changes in the intensity of hyperglycemia therapy. As such, providing evidence-based recommendations on the management of hyperglycemia in patients with different subtypes of CS after curative procedure is challenging.

The goal of our study was to determine the effect of curative procedure on the extent of hyperglycemia and its management in patients with CS. We further aimed to determine the baseline factors (such as CS type, severity, duration of CS, type of hyperglycemia) that are associated with the improvement of hyperglycemia at follow-up.

## Materials and Methods

**Figure 1.**
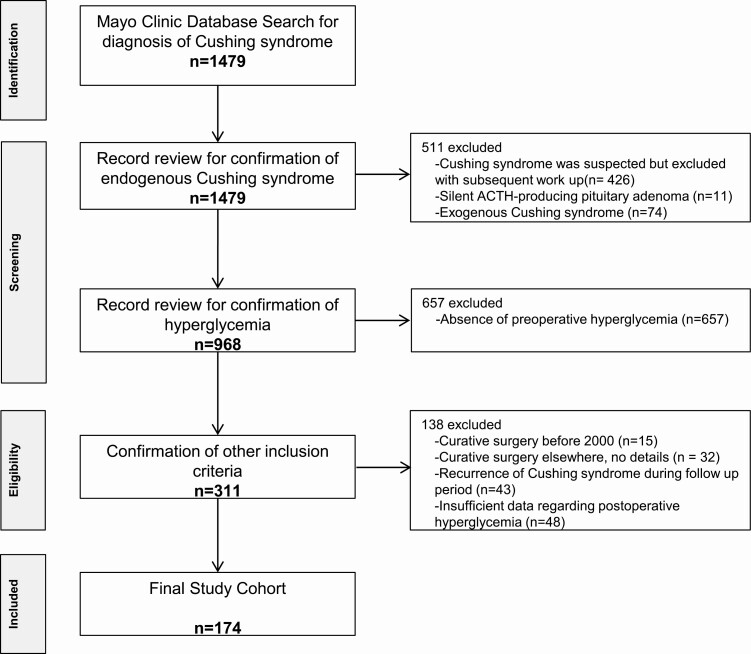
Flowchart for inclusion of patients in the study. The initial search revealed 1479 patients at Mayo with the possible diagnosis of Cushing syndrome (CS). The medical record was reviewed to confirm the diagnosis of endogenous CS and hyperglycemia. The final cohort was determined based on patients who met our inclusion/exclusion criteria.

This retrospective cohort study was approved by the Mayo Clinic Institutional Review Board and included adult patients diagnosed with CS with concurrent hyperglycemia treated with a curative procedure between January 1, 2000, and November 1, 2019. Patient consent waiver was used for this project excluding those who did not give research authorization. Patients were included if they were diagnosed with endogenous CS based on clinical evaluation by the endocrinologist and diagnosis of CS according to the most recent guidelines [1]. Hyperglycemia was classified as DM and prediabetes (impaired glucose tolerance or impaired fasting glucose) diagnosed based on the American Diabetes Association guidelines [23]. All included patients had undergone a curative procedure for treatment of CS (transsphenoidal resection of pituitary adenoma, unilateral or bilateral adrenalectomy, resection of ectopic ACTH-secreting tumor, radiation therapy) and demonstrated remission from CS (defined as low cortisol postoperatively in all patients except in those undergoing bilateral adrenalectomy). Patients needed to have at least one available assessment for hyperglycemia during CS remission. Because patients develop adrenal insufficiency and glucocorticoid withdrawal syndrome following the curative procedure for CS that may last months or be permanent, we have not prespecified a specific required glucocorticoid dose at the end of follow-up, but performed a subgroup analysis based on hydrocortisone dose. We have used the 2 arbitrary cutoffs of 10 and 12 mg of hydrocortisone/m^2^ body surface area for subgroup analysis. Eligible patients were identified through review of medical records of patients with the diagnosis of CS (Fig. 1).

Medical records were reviewed and data on patient demographics, signs, and symptoms of CS, laboratory data, imaging data, the subtype of CS (pituitary, ectopic, adrenal) were collected. Based on the available clinical and laboratory data, each patient was assigned a biochemical and clinical severity of CS classification score, as previously described [24] (Supplementary Tables 1 and 2 [25]).

Data on hyperglycemia included biochemical data (fasting glucose, glycated hemoglobin A_1c_ [HbA_1c_]) and medications used for management of DM. Data were collected before the curative procedure (baseline), and at any available postoperative time points for each individual patient. We collected hyperglycemia data during glucocorticoid taper, with the final postoperative workup when the patient was off glucocorticoids completely, or on stable glucocorticoid chronic replacement therapy after bilateral adrenalectomy.

Our primary end point was improvement or resolution of hyperglycemia following remission of CS with subgroup analyses based on the CS subtype. Resolution of hyperglycemia was defined as the absence of hyperglycemia (based on HbA_1c_ and/or fasting glucose, as well as review of the patient’s glucose log at the end of follow-up), without any need for antihyperglycemic therapy. Improvement was defined as one of the following: 1) improvement in HbA_1c_ and/or fasting glucose concentrations at the end of follow-up while on stable antihyperglycemic therapy; or 2) the decision to decrease the dose and/or number of antihyperglycemic medications/insulin by the treating physician from whom quantitative data were not available (eg, provider notes commenting on glucometer measurements and/or other clinical parameters used to make this judgment).

Secondary end points included changes in HbA_1c_ (Δ HbA_1c_) and the intensity of hyperglycemia management (number of oral medications, total dose of insulin) during follow-up compared to baseline. Subgroup analysis for Δ HbA_1c_ included 1) patients on stable management for hyperglycemia, 2) patients who needed a decrease in the intensity of hyperglycemia management, and 3) patients who needed an increase in the intensity of hyperglycemia management during the follow-up that followed the curative procedure.

### Statistical Analysis

Data were analyzed using JMP 14.1.0 software (SAS Inc). All continuous data were summarized as median and ranges, while categorical data are summarized as a number (percentage). Associations between variables were assessed using the Kruskal-Wallis test for continuous variables and the chi-square test for categorical variables, as appropriate. Multivariable analysis was performed to determine independent predictors of improvement in hyperglycemia. Intraindividual differences were assessed using the matched pair analysis for continuous variables. Statistical significance was defined as *P* less than .05.

## Results

### Patients

Of the 174 patients meeting the inclusion criteria, the median age of diagnosis with CS was 51 years (range, 16-82 years), and 127 (73%) were women. Diagnosis was pituitary CS in 106 patients (60.9%), ectopic CS in 25 patients (14.4%) and adrenal CS in 43 patients (24.7%) (Table 1). Patients with pituitary CS patients were younger at the time of surgery (median age 50 years vs 51 and 56 years in ectopic and adrenal CS; *P* = .001) and included more women (81% vs 48% and 67% in ectopic and adrenal CS; *P* = .002). Patients with pituitary CS also reported the longest duration of CS-related symptoms before diagnosis (median of 36 months vs 12 and 18 months in ectopic and adrenal CS; *P* ≤ .001) (see Table 1). Whereas the biochemical severity score was highest in patients with ectopic CS (severe: 76% vs 59% in pituitary CS and 23.3% in adrenal CS; *P* ≤ .001), the clinical severity score was highest in patients with pituitary CS (severe: 72% vs 60% and 25.6% in ectopic and adrenal CS; *P* ≤ .001) (see Table 1, Supplementary Tables 3 and 4 [25]).

**Table 1.** Clinical and biochemical presentation of patients with Cushing syndrome at time of diagnosis and after remission

Variable	Total N = 174	Pituitary CS N = 106	Ectopic CS N = 25	Adrenal CS N = 43	P
**Age at diagnosis**, y, median (range)	51 (16-82)	49 (16-82)	51 (34-77)	55 (35-78)	< .001
**Age at time of curative procedure**, y, median (range)	52.5 (19-82)	50 (19-82)	51 (34-78)	56 (37-78)	.001
**Women,** n (%)	127 (73.0%)	86 (81.1%)	12 (48%)	29 (67.4%)	.002
**BMI**, median (range) Available for n = 170	34.84 (21.03-66.4)	36.97 (22.4-66.4)	29.53 (21-41.6)	32.7 (22-48.4)	< .001
**CS biochemical severity score**, n (%)					< .001
Mild	25 (14.5%)	8 (7.6%)	4(16%)	13 (30.2%)	
Moderate	57 (32.9%)	35 (33.3%)	2 (8%)	20 (46.5%)	
Severe	91 (52.6%)	62 (59.1%)	19 (76%)	10 (23.3%)	
Available for n = 173					
**CS clinical severity** score, n (%)					< .001
Mild	16 (9.2%)	3 (2.8%)	2 (8%)	11 (25.6%)	
Moderate	56 (32.2%)	27 (25.5%)	8 (32%)	21 (48.8%)	
Severe	102 (58.6%)	76 (71.7%)	15 (60%)	11 (25.6%)	
**Duration of hypercortisolism before curative procedure**, mo, median (range)	24 (0-240)	36 (0-240)	12 (0.5-108)	18 (0-228)	< .001
Biochemical assessment					
**24-h urine cortisol,** mcg/24 h Median (range) Available for n = 158 Normal range: < 45 mcg/24 h	180.5 (5.7-6459)	178.5 (7.7-2638)	990 (133-6459)	73 (5.7-1643)	< .001
**1-mg dexamethasone suppression test,** mcg/dL Median (range) Available for n = 103 Normal range: < 1.8 mcg/dL	13.7 (2.2-85.4)	17 (3.8-78.5)	51.35 (8-85.4)	6.35 (2.2-60)	< .001
**ACTH,** pg/mL, median (range) Available for n = 169 Normal range: 6-63 pg/mL	73.5 (2-531)	81 (21-387)	185 (29-531)	5.7 (2-40)	< .001
**Salivary cortisol,** ng/dL, median (range) Available for n = 55 Normal range: < 50 ng/dL	263 (41-8300)	265.5 (50-1600)	2550 (645-8300)	109 (41-1270)	.002
**Curative procedure for CS**					
Pituitary surgery, n (%)	77 (44.3%)	77 (72.6%)	–	–	
Pituitary radiation^*a*^, n (%)	2 (1.1%)	2 (1.9%)	–	–	
Resection of the neuroendocrine tumor, n (%)	10 (5.7%)	–	10 (40%)	–	
Unilateral adrenalectomy, n (%)	36 (20.7%)	–	–	36 (83.7%)	
Bilateral adrenalectomy, n (%)	49 (28.2%)	27 (25.5%)	15 (60%)	7 (16.3%)	
**Postoperative adrenal insufficiency and glucocorticoid replacement therapy after curative procedure**					
**Patients taking glucocorticoids at end of follow-up**, n (%)	72 (41.4%)	47 (44.3%)	10 (40%)	15 (34.9%)	.08
Duration of supraphysiological glucocorticoid replacement therapy, mo, median (range)^*b*^	6.5 (0-45)	8 (0-23)	2 (0-30)	7 (1-24)	
**Recovery of adrenal function at end of follow-up**, n (%)	102 (58.6%)	59 (55.7%)	15 (60%)	28 (65.1%)	.38
Duration of glucocorticoid replacement therapy, mo, median (range)^*c*^	13 (0-139)	13 (1-139)	10 (0-65)	14.5 (0-55)	

Abbreviations: ACTH, adrenocorticotropin; BMI, body mass index; CS, Cushing syndrome.

^*a*^Previously had pituitary surgery. Biochemical recurrence was noted, leading patients to treatment before symptom recurrence (no medical therapy required). Evidence of improvement after radiation was documented biochemically.

^*b*^Defined as patients who were still on glucocorticoid replacement or patients with bilateral adrenalectomy on more than 30-mg hydrocortisone equivalent.

^*c*^Two patients with adrenal CS were on glucocorticoids for less than 1 month (mild CS or rapid improvement of hypothalamic-pituitary-adrenal axis), and one patient with ectopic CS was on ketoconazole preoperatively, which contributed to short duration of steroids.

### Hyperglycemia at the Time of Diagnosis

At the time of CS diagnosis, patients presented with type 2 DM (139 patients, 79.9%), prediabetes (33, 19.0%), and type 1 DM (2, 1.1%). Duration of hyperglycemia before presentation with CS was highest in adrenal CS (median of 21 months vs median of 19 and 1 month in pituitary and ectopic CS; *P* = .02) (Table 2). Patients with pituitary CS were more likely to have DM vs prediabetes when compared to patients with ectopic and adrenal CS (86.8% vs 68.0% and 69.8%; *P* = .03) (see Table 2). At baseline, fasting glucose and HbA_1c_ were similar in CS subtypes; however, the proportion of patients treated for hyperglycemia was highest in patients with pituitary CS (86% vs 76% in ectopic CS and 58.2% in adrenal CS; *P* = .003). A higher number of patients with pituitary CS were treated with oral medications (62.3% vs 32.0% in ectopic 44.2% in adrenal; *P* = .04) (see Table 2). No differences in the proportion of patients treated with insulin or in the daily insulin dose were observed in CS subtypes.

**Table 2.** Characterization of hyperglycemia management in patients with Cushing syndrome at time of diagnosis and after remission

Variable	Total N = 174	Pituitary CS N = 106	Ectopic CS N = 25	Adrenal CS N = 43	P
**Baseline diagnosis**					
**Duration of hyperglycemia at time of diagnosis with CS**, mo, median (range) Available for n = 164	16 (0-458)	19.5 (0-249)	1 (0-190)	21 (0-458)	.02
**Type of hyperglycemia,** n (%)					
DM2	139 (79.9%)	92 (86.8%)	17 (68%)	30 (69.8%)	.03
IGT/IFG/Prediabetes	33 (19.0%)	13 (12.3%)	7 (28%)	13 (30.2%)	
DM1	2 (1.1%)	1 (0.9%)	1 (4%)	0 (0%)	
**Baseline HbA**_**1c**_, %, median (ranges) Available for n = 165	6.9 (4.9-13.1)	7.1 (4.9-13.1)	6.75 (5.3-11.4)	6.6 (5.4-11.1)	.35
**Baseline fasting glucose,** mg/dL, median (ranges) Available for n = 168	132 (60-427)	135 (60-427)	133 (90-368)	130 (80-330)	.40
**Baseline hyperglycemia therapy** ^*b*^					
No therapy, n (%)	41 (23.6%)	17 (16.0%)	6 (24.0%)	18 (41.8%)	.003
Patients treated with oral medications^*a*^, n (%) No. of oral medications, n (%)	93 (53.4%)	66 (62.3%)	8 (32.0%)	19 (44.2%)	.04
1	59 (33.9%)	39 (36.8%)	6 (24.0%)	14 (32.6%)	
2	26 (14.9%)	19 (17.9%)	2 (8.0%)	5 (11.6%)	
3	8 (4.6%)	8 (7.5%)	0 (0%)	0 (0%)	
Patients treated with insulin, n (%)	64 (36.8%)	39 (36.8%)	12 (48.0%)	13 (30.2%)	.34
Insulin daily units, median (ranges)	58 (10-360)	75 (15-360)	32.5 (12-155)	48 (10-116)	.06
**Hyperglycemia during postoperative period, final workup**					
Resolution of hyperglycemia, n (%)	37 (21.3%)	22 (20.8%)	9 (36%)	6 (14.0%)	.001
Improvement of hyperglycemia, n (%)	82 (47.1%)	60 (56.6%)	9 (36%)	13 (30.2%)	
Absence of improvement of hyperglycemia, n (%)	55 (31.6%)	24 (22.6%)	7 (28%)	24 (55.8%)	
**Follow-up HbA**_**1c**_ **, final workup** %, median (ranges) Available for n = 130	6.1 (4.4-11.3)	6.0 (4.4-10.3)	5.9 (4.5-8.1)	6.4 (5.2-11.3)	.20
**Follow-up fasting glucose, final workup,** mg/dL, median (ranges) Available for n = 141	108 (63-270)	106 (69-224)	97 (82-152)	112 (63-270)	.11
**Follow-up hyperglycemia therapy, final workup** ^*c*^					
No therapy, n (%)	80 (46.0%)	43 (40.6%)	16 (64.0%)	21 (48.8%)	.10
Patients treated with oral medications^*a*^, n (%) No. of oral medications, n (%)	60 (34.5%)	42 (39.6%)	4 (16.0%)	14 (32.6%)	.30
1	44 (25.3%)	30 (28.3%)	4 (16.0%)	10 (23.3%)	
2	13 (7.5%)	9 (8.5%)	0 (0%)	4 (9.3%)	
3	3 (1.7%)	3 (2.8%)	0 (0%)	0 (0%)	
Patients treated with insulin, n (%)	42 (24.1%)	24 (22.6%)	7 (28.0%)	11 (25.6%)	.83
Insulin daily units, median (ranges)	63 (7-236)	73 (12-236)	40 (7-96)	38 (15-125)	.24

Abbreviations: CS, Cushing syndrome; DM, diabetes mellitus; HbA_1c_, glycated hemoglobin A_1c_; IFG, impaired fasting glucose; IGT, impaired glucose tolerance.

^*a*^Owing to small sample size, use of noninsulin injectables was not included.

^*b*^Patients treated with orals alone, n = 68, insulin alone n = 39, and both orals/insulin n = 25.

^*c*^Patients treated with orals alone, n = 44, insulin alone n = 26, both orals/insulin n = 16.

In patients treated with oral medications (n = 93), metformin was the most used (n = 81, 87.1%), followed by sulfonylurea drugs (n = 31, 33.3%) (Supplementary Table 5 [25]). Other noninsulin agents used include thiazolidinediones (n = 13, 14.0%), glucagon-like peptide-1 agonists (n = 9, 9.7%), sodium-glucose cotransporter-2 inhibitors (n = 6, 6.5%), and dipeptidyl peptidase-IV inhibitors (n = 5, 5.4%). In patients on one or more types of insulin (n = 64), basal insulin was used in 48 patients (75.0%) and rapid-acting insulin in 39 patients (60.9%). Other insulins used included intermediate-acting (n = 10, 15.6%), premixed (n = 3, 4.7%), regular (n = 2, 3.1%), insulin pumps (n = 2, 3.1%), and U-500 insulin (n = 1, 1.6%) (see Supplementary Table 5 [25]).

### Perioperative Management of Hyperglycemia

At the time of surgery, an inpatient specialist diabetes consulting service was used in the care of 97 patients (55.7%), more commonly if patients had ectopic CS, in those with more severe hyperglycemia, and those treated with insulin (Table 3).

**Table 3.** Use of inpatient specialist consulting service

Variable	Consulting service involved N = 97	Consulting service not involved N = 77	P
**CS subtype, n (%)**			.005
Pituitary	60 (61.9%)	46 (59.7%)	
Ectopic	20 (20.6%)	5 (6.5%)	
Adrenal	17 (17.5%)	26 (33.8%)	
**Biochemical severity score, n (%)**			.14
Mild	12 (12.5%)	13 (16.9%)	
Moderate	27 (28.1%)	30 (39.0%)	
Severe	57 (59.4%)	34 (44.1%)	
Available for n = 173			
**Clinical severity score, n (%)**			.22
Mild	9 (9.3%)	7 (9.1%)	
Moderate	26 (26.8%)	30 (39.0%)	
Severe	62 (63.9%)	40 (51.9%)	
**Hyperglycemia diagnosis, n (%)**			.06
DM2	82 (84.5%)	57 (74.0%)	
DM1	2 (2.1%)	0 (0%)	
IGF/IGT/Prediabetes	13 (13.4%)	20 (26.0%)	
**Presurgical HbA**_**1c**_ **(%), median (range)**	7.2 (5.3-13.1)	6.6 (4.9-10.7)	< .001
**Planned surgical procedure, n (%)**			.08
Pituitary surgery	45 (46.4%)	32 (41.5%)	
Pituitary radiation	0 (0%)	2 (2.6%)	
Resection of neuroendocrine tumor	7 (7.2%)	3 (3.9%)	
Unilateral adrenalectomy	15 (15.5%)	21 (27.3%)	
Bilateral adrenalectomy, n (%)	30 (30.9%)	19 (24.7%)	
**No. of noninsulin agents, n (%)**			.18
0	44 (45.3%)	36 (46.7%)	
1	35 (36.1%)	19 (24.7%)	
2	12 (12.4%)	18 (23.4%)	
3	6 (6.2%)	4 (5.2%)	
**Insulin**			
On insulin preadmission, n (%)			< .001
Yes	50 (51.6%)	14 (18.2%)	
No	47 (48.4%)	63 (81.8%)	
Units of insulin, median (range)	54 (10-360)	92 (12-206)	.6

Abbreviations: CS, Cushing syndrome; DM, diabetes mellitus; HbA_1c_, glycated hemoglobin A_1c_; IFG, impaired fasting glucose; IGT, impaired glucose tolerance.

Twelve patients (6.9%) had a significant program change that intensified their discharge DM regimen (eg, adding insulin, new oral medications); the specialist consulting service was involved in 11 of these cases (91.7%). Other changes of intensified pharmacotherapy noted at discharge included 5 patients (2.9%) who had their noninsulin medication dosage(s) increased, and 5 patients (2.9%) who had their insulin doses increased. All other patients did not have a program change (whether on medications or not) or had their medication dosage(s) reduced at the time of discharge.

### Hyperglycemia After Curative Procedure for Cushing Syndrome

Patients were followed for hyperglycemia management for a median of 10.5 months (range, 0-130 months) after achieving CS remission. At the time of final follow-up, 102 patients (58.6%) were either no longer treated with glucocorticoids or reached a stable glucocorticoid replacement dose. Median hydrocortisone mg/m^2^ body surface area was 8.1 (interquartile range = 0-13.4) at the time of final follow-up.

**Figure 2.**
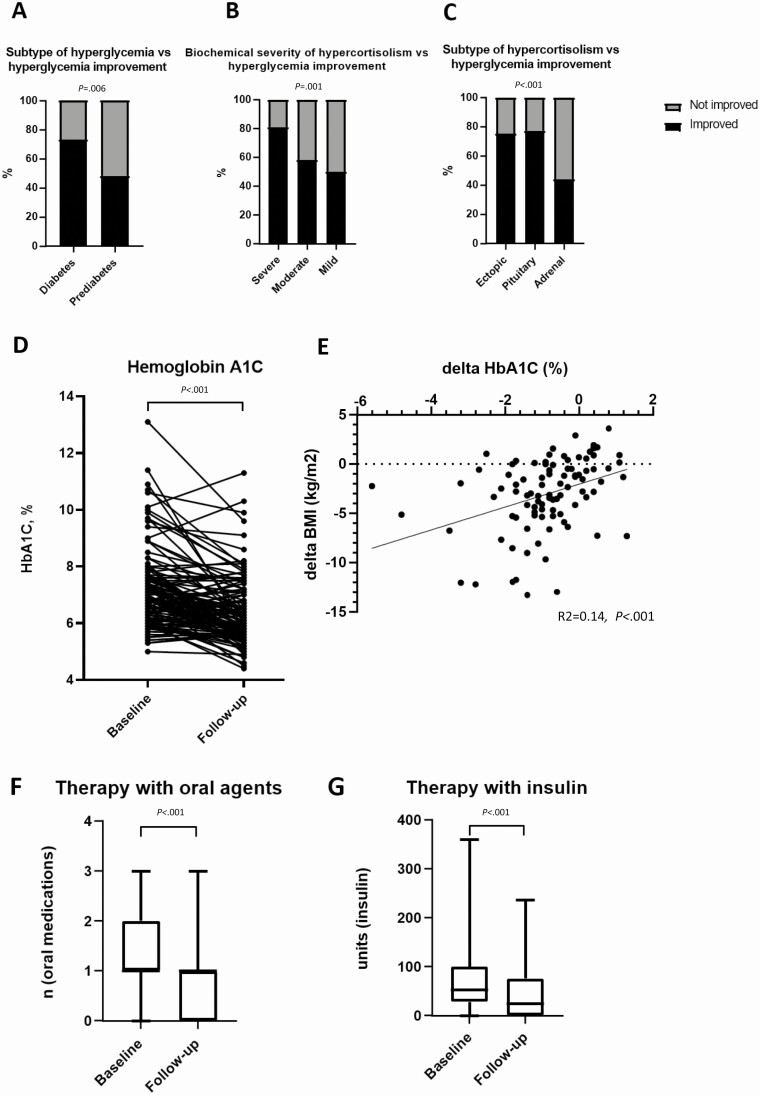
Baseline and postoperative glycated hemoglobin A_1c_ (HbA_1c_), number of oral hyperglycemic medications, and total daily insulin units. A, Improvement of hyperglycemia based on the subtype of hyperglycemia. B, Improvement of hyperglycemia based on the biochemical severity of hypercortisolism. C, Improvement of hyperglycemia based on the subtype of hypercortisolism. D, HbA_1c_ at baseline and at follow up (n = 130). E, Association of the Δ HbA_1c_ with Δ body mass index following the curative procedure for Cushing syndrome. F, Number of oral medications at baseline and at follow-up in 93 patients initially treated with oral medications for hyperglycemia. G, Total daily insulin units at baseline and at follow up (n = 64).

Subtype of hyperglycemia, biochemical severity of hypercortisolism, and subtype of hypercortisolism were associated with improvement of hyperglycemia during follow up (Fig. 2A-2C). When compared to baseline, at the time of final follow-up, mean HbA_1c_ had decreased by a mean of 0.84%; *P* ≤ .001 (Fig. 2D). The degree of HbA_1c_ decrease (Δ HbA_1c_) was associated with weight loss (Δ body mass index [BMI]) (Fig. 2E). In 60 patients treated with one or more noninsulin agents at baseline, the overall number of medications decreased at follow-up by a mean of 0.5 agents (from mean of 1.3 to mean of 0.76, *P* ≤ .001) (Fig. 2F). In 42 patients treated with insulin at baseline, the mean total daily insulin use decreased by a mean of 30 units (*P* ≤ .001) Fig. 2G). In 33 patients treated with insulin at baseline and follow-up, the mean total daily insulin/kg dose decreased by –0.19 units/kg/day (95% CI, –0.02 to –0.36) (*P* = .02).

**Figure 3.**
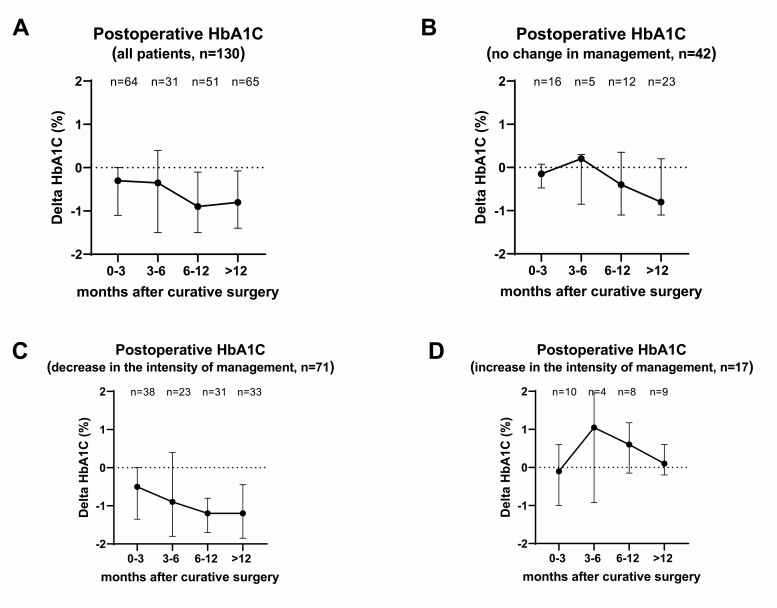
Δ Glycated hemoglobin A_1c_ (HbA_1c_) following the curative procedure for Cushing syndrome. A, Δ HbA_1c_ in all patients (n = 130). B, Δ HbA_1c_ in 42 patients in whom hyperglycemia management was not changed (including those on no medications). C, Δ HbA_1c_ in 71 patients in whom the intensity of hyperglycemia management was decreased (decrease in dose or number of medications). D, Δ HbA_1c_ in 17 patients in whom the intensity of hyperglycemia management was increased (increase in dose or number of medications, new start of medications).

At the time of final follow-up, hyperglycemia improved in 118 patients (68.6%) (Table 4). Overall, HbA_1c_ decreased gradually during follow-up (Fig. 3A). In patients in whom no changes in therapy for hyperglycemia during follow-up was instituted (n = 42), HbA_1c_ decreased by a mean of –0.5% (median of –0.3%) by the end of follow-up (Fig. 3B). In 71 patients in whom the intensity of hyperglycemia management had to be decreased during follow-up, HbA_1c_ decreased by a mean of –1.2% (median of –1.0%) (Fig. 3C). Only 17 patents had to intensify hyperglycemia management after curative surgery and demonstrated a mean Δ HbA_1c_ of +0.2% (median of +0.1%) (Fig. 3D). This cohort consisted of 9 patients with pituitary CS (52.9%), 7 patients with adrenal CS (41.2%), and 1 patient with ectopic CS (5.9%). Of these 17 patients, 5 (29.4%) had severe CS, and baseline median HbA_1c_ was 6.9% (5.5%-10.7%). Univariate and multivariable analysis did not demonstrate a significant factor in this group that was associated with the need to increase therapy (data not shown). Notably, no differences in the initial postoperative daily glucocorticoid dose, in the number of patients still treated with glucocorticoids, or in the median daily hydrocortisone dose at the end of follow-up were found between the groups based on the intensity of hyperglycemia management (Supplementary Table 6 [25]).

In a subgroup analysis of patients who discontinued glucocorticoid therapy at the last follow-up compared to patients who were still being treated with glucocorticoids, or based on the final daily hydrocortisone cutoff of 10 and 12 mg/m^2^ body surface area, no differences in the decrease of HbA_1c_ or the proportion of patients with improved hyperglycemia were observed (Supplementary Table 7 and 8 [25]). In another subgroup analysis of patients treated with bilateral adrenalectomy compared to patients treated with other procedures, no differences in the decrease of HbA_1c_ or proportion of patients with improvement of hyperglycemia were found (Supplementary Table 9 [25]).

On univariate analysis, age, sex, and BMI were not associated with improvement of hyperglycemia following CS remission (Table 4). In contrast, higher severity of CS based on the biochemical and clinical severity scores, ACTH-dependent vs -independent CS, and DM2 vs prediabetes were associated with improvement of hyperglycemia (see Table 4 and Fig. 2A-2C). Severity of CS (OR = 2.4; 95% CI, 1.1-4.9) and ACTH-dependent CS (OR = 2.9; 95% CI, 1.3-6.4), but not the type of hyperglycemia (DM vs prediabetes: OR = 2.13 [95% CI of 0.9-4.9]), were found to be associated with hyperglycemia improvement on multivariable analysis (see Table 4).

**Table 4.** Factors associated with improvement in hyperglycemia after curative procedure*^a^*

Univariate analysis				Multivariable analysis		
Variable	Improvement in hyperglycemia N = 118	No improvement in hyperglycemia N = 54	*P*	Variable	Odds ratio (95% CI)	P
**Age, y,** median (range)	52.5 (19-82)	54 (28-78)	.34			
**Female sex**, n (%)	85 (72%)	41 (75.6%)	.59			
**BMI,** median (range)	34.8 (21.0-66.4)	37 (22.0-53.8)	.76			
**Duration of hypercortisolism, mo,** median (range)	18 (0-240)	12 (0-204)	.49			
**Duration of hyperglycemia, mo,** median (range)	15.5 (0-350)	25 (0-458)	.36			
**Biochemical severity score**, n (%)						
Mild	12 (10.1%)	12 (22.6%)	.001	**Biochemical severity score** ^*b*^ (severe vs other)	2.4 (1.1-4.9)	.02
Moderate	33 (28%)	24 (45.3%)				
Severe	73 (61.9%)	17 (30.1%)				
Available for n = 173						
**Clinical severity score**, n (%)						
Mild	6 (5.1%)	9 (16.7%)	.001			
Moderate	31 (26.3%)	24 (44.4%)				
Severe	81 (68.6%)	21 (38.9%)				
**Type of CS**, n (%)						
Adrenal	19 (16.1%)	24 (44.4%)	< .001	**Type of CS** (ACTH-dependent vs adrenal)^*c*^	2.9 (1.3-6.4)	.007
Ectopic	18 (15.3%)	6 (11.2%)				
Pituitary	81 (68.6%)	24 (44.4%)				
**Hyperglycemia diagnosis**, n (%)						
Prediabetes	16 (13.6%)	17 (31.5%)	.006	**Hyperglycemia diagnosis** (diabetes vs prediabetes)	2.13 (0.9-4.9)	.08
DM2	102 (86.4%)	37 (68.5%)				

Abbreviations: ACTH: adrenocorticotropin; BMI, body mass index; CS, Cushing syndrome; DM, diabetes mellitus.

^*a*^Patients with DM1 were excluded (n = 2).

^*b*^Because biochemical and clinical severity scores were concordant, only one was chosen for the multivariable analysis.

^*c*^Pituitary and ectopic CS were combined into ACTH-dependent subtype for the multivariable analysis.

## Discussion

In this study, we characterized the presentation at baseline and the evolution of hyperglycemia in patients with CS following the curative procedure and CS remission. We showed that two-thirds of patients demonstrated resolution or improvement of hyperglycemia during follow-up, and more than half were able to decrease the dose or the number of medications used for hyperglycemia. Patients with ACTH-dependent CS and those with a higher biochemical severity score were more likely to have improved hyperglycemia during follow-up.

We found that at the time of diagnosis, no differences in baseline HbA_1c_ or fasting glucose were found between the CS subtypes; however, patients with pituitary CS were more commonly treated for hyperglycemia and with a higher number of hyperglycemia agents than patients with other CS subtypes. Potential explanations for this finding include a higher duration of hypercortisolism in patients with pituitary CS before diagnosis, along with a high proportion of patients with severe biochemical and clinical CS severity scores. Patients with pituitary CS also had the highest BMI, further contributing to the severity of hyperglycemia at baseline. While several studies reported on the differences in the prevalence of hyperglycemia among CS subtypes [2, 4, 6, 15], none commented on the severity and the intensity of therapy of hyperglycemia at the time of diagnosis based on CS subtype.

We found that almost half of patients in our study demonstrated improvement in hyperglycemia, and another 21% experienced resolution of hyperglycemia following the curative procedure for CS. Several small studies in patients with CS reported resolution of hyperglycemia in 20% to 79% of patients [14-17, 19-22]. Improvement in hyperglycemia following adrenalectomy was reported in 52% of patients with mild autonomous cortisol secretion [26], but was not systematically reported for patients with CS. In our study we defined improvement as a decrease in HbA_1c_ and/or a decrease in the dose or number of medications used to treat hyperglycemia. Applying this definition, 47% of our patients demonstrated improvement in hyperglycemia by the end of follow-up. We showed that the overall HbA_1c_ decreased by 0.84%, and only a minority of patients needed to intensify their hyperglycemia regimen after achieving CS remission.

We identified several factors that were associated with either resolution or improvement of hyperglycemia. These included the severity of CS (both clinical and biochemical), type of CS, and severity of hyperglycemia at the time of diagnosis. ACTH-dependent CS and severe biochemical score were independent predictors of hyperglycemia improvement on multivariable analysis. These data suggest that in patients with milder forms of CS, hypercortisolism may play a smaller role in hyperglycemia at the time of diagnosis, and a subsequent curative procedure is less likely to improve it.

Several clinical implications arise from our study. First, as improvement in hyperglycemia is seen in most patients, counseling and close monitoring of those treated with insulin or other medications is needed to modify therapy and avoid hypoglycemia. Second, patients with milder CS (more commonly adrenal) should be treated for more severe hyperglycemia rather than adopt an expectant attitude because hyperglycemia in these cases is less likely to improve with CS remission. Notably, not all patients achieved an optimal glucose control during follow-up, suggesting that intensification of therapy rather than an expectant attitude is warranted in some patients with CS.

Our study has several strengths. We include a large sample size, with appropriate patient representation regarding CS subtype and severity, and type of hyperglycemia. Availability of a detailed medical record system allowed us to perform an in-depth analysis both of baseline and follow-up variables. This is the first study to analyze the quantitative changes, based on the time from the curative surgery, to assess the changes in the intensity of hyperglycemia therapy and identify predictors for hyperglycemia improvement. The limitations of our study include the retrospective design with selection, referral, and information biases. Not all patients with CS treated at our institution were followed for hyperglycemia after surgery for the whole duration of follow-up. Management of hyperglycemia was not standardized and differed among clinicians. While the initial diagnosis of hyperglycemia was made based on the American Diabetes Association guidelines, resolution of hyperglycemia was based on multiple factors, such as glucose log, normal fasting glucose, and HbA_1c_ measurements in a patient not treated with pharmacotherapy for hyperglycemia. Duration of follow-up varied, and at the time of last follow-up not all patients recovered adrenal function. Some of the subgroup analyses were limited by the small sample size.

In conclusion, we demonstrated that almost 70% of patients with CS demonstrate either resolution or improvement in hyperglycemia following CS remission. As a group, patients demonstrate a decrease in HbA_1c_, and can be treated with less insulin and fewer noninsulin agents. Patients with more severe hyperglycemia, ACTH-dependent CS, and more severe CS are more likely to improve after surgery. Patients with hyperglycemia need appropriate monitoring and medication adjustment after surgery for CS.

## Data Availability

Some or all data sets generated during and/or analyzed during the present study are not publicly available but are available from the corresponding author on reasonable request.

## Abbreviations

ACTH: adrenocorticotropin
BMI: body mass index
CS: Cushing syndrome
DM: diabetes mellitus
HbA_1c_: glycated hemoglobin A_1c_
OR: odds ratio
